# Clinical characteristics and prognostic factors of adult patients with pilocytic astrocytoma

**DOI:** 10.1007/s11060-020-03513-9

**Published:** 2020-04-27

**Authors:** Maximilian J. Mair, Adelheid Wöhrer, Julia Furtner, Anika Simonovska, Barbara Kiesel, Stefan Oberndorfer, Karl Ungersböck, Christine Marosi, Felix Sahm, Johannes A. Hainfellner, Karl Rössler, Matthias Preusser, Georg Widhalm, Anna S. Berghoff

**Affiliations:** 1 Division of Oncology, Department of Medicine I, Medical University of Vienna, Vienna, Austria; 2 Division of Neuropathology and Neurochemistry, Department of Neurology, Medical University of Vienna, Vienna, Austria; 3 Department of Radiology and Biomedical Imaging, Medical University of Vienna, Vienna, Austria; 4 Department of Neurosurgery, Medical University of Vienna, Waehringer Guertel 18-20, 1090 Vienna, Austria; 5 Department of Neurology, University Clinic of St. Pölten, St. Pölten, Austria; 6 Department of Neurosurgery, University Clinic of St. Pölten, St. Pölten, Austria; 7 Department of Neuropathology, Institute of Pathology, Ruprecht-Karls University Heidelberg, and Clinical Cooperation Unit Neuropathology, German Consortium for Translational Cancer Research (DKTK), German Cancer Research Center (DKFZ), Heidelberg, Germany

**Keywords:** Pilocytic astrocytoma, Adult, Low-grade glioma, Primary CNS tumor

## Abstract

**Introduction:**

Pilocytic astrocytoma (PA) is the most common primary brain neoplasm in children and treated in curative intent with gross total resection (GTR). However, PA is rare in adults, resulting in limited knowledge on the natural clinical course. This study aimed to describe the clinical course and identify prognostic factors of adult patients with PA.

**Methods:**

46 patients ≥ 18 years at diagnosis of PA and neurosurgical resection or biopsy between 2000 and 2018 were identified from the Neuro-Biobank of the Medical University of Vienna. In two cases with differing histopathological diagnosis at recurrence, DNA methylation analysis was performed using Illumina Infinium HumanMethylation850 BeadChip (850 k) arrays and the Molecular Neuropathology classifier. Clinico-pathological features were correlated with patient outcomes.

**Results:**

Median age at diagnosis was 32.5 years (range: 19–75) and median Ki67 proliferation index was 2.8% (0.5–13.4%). Tumor location significantly correlated with resectability (p < 0.001). Tumor progression or recurrence was observed in 9/46 (19.6%) patients after a median follow up time of 53.0 months (range 0.5–300). 5-year overall and progression-free survival rates were 85.3% and 70.0%, respectively. 2/9 (22.2%) patients presented with histological changes in the recurrent tumor specimen. In detail, methylation classification redefined the histological diagnosis to anaplastic astrocytoma with piloid features and glioma in one patient, each. Age > 40 and higher body mass index (BMI) were associated with impaired progression-free and overall survival (p < 0.05).

**Conclusions:**

Tumor recurrence or progression in adult PA patients was higher than the one reported in pediatric patients. Higher age and BMI were associated with impaired prognosis.

## Introduction

Pilocytic astrocytomas (PA) occur with an age-adjusted incidence rate of 0.91/100,000 per year and are the most common primary central nervous system tumor in children and adolescents [1]. In young adults, the incidence declines as PA account for only 0.8% of CNS tumors in patients older than 19 years in contrast to 11.4% in 0–19 years-old patients [1]. In the current WHO classification of Central Nervous Tumours, PA are classified as grade I tumors with a characteristically well-circumscribed growth pattern [2]. In consequence, gross total resection (GTR) is the main treatment approach to achieve cure in pediatric as well as adult patients. 10-year overall survival after GTR reaches more than 90% in the pediatric population [3]. So far only limited data on the prognostic assessment of adult PA patients exists, as most series were rather small and frequently did not include patients aged > 50 years. Indeed, the prognostic value of GTR, age and locations was controversially discussed based on previously published small series and population-based studies [4–7]. In line, the application of adjuvant therapy approaches including radiation in case of incomplete resection was a matter of debate in adult PA patients. Further insights on prognostic assessment in adult PA patients are needed to ensure precise selection. Therefore, we aimed to analyze a broader pattern of clinical factors and the association with clinical course in a large adult PA cohort treated at a tertiary care center.

## Methods

### Patient cohort

Patients aged ≥ 18 years at diagnosis and histological confirmation of pilocytic (PA) or pilomyxoid astrocytoma (PMA) and treated between 2000 and 2018 were identified from the Neuro-Biobank of the Medical University of Vienna. Histological diagnosis was confirmed by a board-certified neuropathologist. Clinical data were collected by chart review. Pre-operative resectability (resectable or unresectable) was estimated from two board-certified neurosurgeons (K.R., G.W.) based on preoperative imaging. Invasion of tumor tissue into neighboring structures was retrieved from surgical reports. The extent of surgical resection was obtained from postoperative imaging. Recorded symptoms included new-onset headache, visual disturbances, symptoms of elevated intracranial pressure (ICP), vertigo, motoric and sensory deficits, psychiatric symptoms such as personality changes or mood-related symptoms, seizures, ataxia and speech disturbances. Highly symptomatic disease was defined by presence of at least 3 of the above-mentioned symptoms. Patient data were collected in a password-secured database (FileMaker Pro® Advanced 17, FileMaker Inc., Santa Clara, CA, USA) and were handled anonymously.

### Tissue-based analysis

Formalin-fixed, paraffin-embedded (FFPE) tumor tissue blocks were cut with a regular microtome for further immunohistochemical and methylation analysis. Immunohistochemistry for Ki67 was performed by an automated slide processing system (Autostainer Plus Link, Dako, Glostrup, Denmark; MIB-1/clone M7240, Dako) as previously described [8]. The Ki67 proliferation index was obtained by counting 500 cells in the most densely stained area and is given as a percentage (0–100%). Methylation analysis was performed of samples with resection at recurrence and differing histological features as compared to the first resection. DNA extracted from FFPE tissue was analyzed as described previously using Illumina Infinium HumanMethylation850 BeadChip (850 k) arrays (Illumina, San Diego, CA, USA) [9]. Samples were classified using the previously published MolecularNeuropathology classifier [10].

### Statistical analysis

Overall survival (OS) was calculated as the time span between first radiological diagnosis and all-cause death or last follow up for patients that were still alive. Progression-free survival (PFS) was defined as the time from first radiological diagnosis to progression/recurrence or all-cause death or last follow up, where the date of progression was retrieved from the first radiological report expressing the suspicion of tumor recurrence or progression. Chi square test and Fisher’s exact test were applied as appropriate. Survival estimates were calculated using the Kaplan Meier method and survival differences between groups were analyzed by applying the log-rank test. Results were considered significant at a p-value of < 0.05. Cases with missing data were excluded from the respective analyses. As the purpose of this exploratory study was the generation of hypotheses, no adjustment for multiple testing was applied [11]. Statistical analysis was performed using IBM SPSS Statistics 25 (IBM, Armonk, NY, USA) and GraphPad Prism 8 for Mac (La Jolla, CA, USA).

## Results

### Patients’ characteristics

46 patients with a median age of 32.5 years (range: 19–75 years) at diagnosis were included in this study. Notably, 7/46 (15.2%) patients were aged 50 years or older at time of diagnosis. 26/46 (56.5%) patients were male, while 20/46 (43.5%) were female, with a resulting male-to-female ratio of 1.3:1. Of note, there were no patients with a confirmed diagnosis of a genetic syndrome such as neurofibromatosis. Table 1 summarizes further patients’ characteristics.

**Table 1:** Baseline and treatment characteristics

	n = 46	%
Gender		
Male	26	56.5
Female	20	43.5
Age at diagnosis (years)		
Median (range)	32.5 (19–75)	
≤ 40	32	69.6
40–49	7	15.2
50–69	5	10.9
≥ 70	2	4.3
Histology		
Pilocytic astrocytoma (WHO Grade I)	44	95.7
Pilomyxoid astrocytoma (WHO Grade II)	2	4.3
Ki67 proliferation index at first diagnosis		
Median (range)	2.8% (0.5–13.4%)	
Karnofsky performance score at presentation		
Median (range)	90% (70–100%)	
BMI at presentation (n = 37)		
Median (range)	22.84 (16.65–46.30)	
Symptoms at presentation		
New-onset headache	30	65.2
Visual disturbances	16	34.8
Symptoms of elevated ICP	13	28.3
Vertigo	13	28.3
Motoric	9	19.6
Sensory	8	17.4
Psychiatric symptoms	6	13.0
Seizures	4	8.7
Ataxia	4	8.7
Speech disturbance	2	4.3
Symptomatic burden at presentation		
≤ 2 symptoms	29	63.0
> 2 symptoms	17	37.0
Tumor location/resectability^a^	34	
Optic nerve with hypothalamic involvement	8	23.5
Resectable	1	
Unresectable	7	
Optic nerve without hypothalamic involvement	4	11.8
Resectable	2	
Unresectable	2	
Cerebral hemispheres	8	23.5
Resectable	8	
Unresectable	0	
Cerebellar hemispheres	6	17.6
Resectable	6	
Unresectable	0	
Cerebellar with brainstem involvement	5	14.7
Resectable	3	
Unresectable	2	
Lower brainstem/spinal	3	8.8
Resectable	0	
Unresectable	3	
Primary resection	46	100.0
Available postoperative imaging	32	69.6
Gross total resection	18	39.1
Subtotal resection	1	2.2
Extended biopsy	5	15.6
Biopsy	8	17.4
Dexamethasone use at first diagnosis/perioperatively		
Yes	23	50.0
No	9	19.6
Unknown	14	30.4
Treatments at first progression	8	17.4
Primary resection	6	13.0
Subtotal resection	5	10.9
Biopsy	1	2.2
Gamma knife radiosurgery	2	4.3
Adjuvant radiochemotherapy	1	2.2
Median follow up in months (range)	53.0 (0.5–300.1)	

*MRI* magnetic resonance imaging

^a^Only for patients with available preoperative imaging

### Symptomatic burden of adult pilocytic astrocytoma patients

Median Karnofsky Performance Scale (KPS) at diagnosis was 90% (range: 70–100%). Most patients presented with new-onset headache (30/46, 65.2%), followed by visual disturbances (16/46, 34.8%), symptoms of elevated ICP (13/46, 28.3%), vertigo (13/46, 28.3%), motoric deficits (9/46, 19.6%), sensory symptoms (8/46, 17.4%), psychiatric symptoms (6/46, 13.3%), seizures (4/46, 8.7%), ataxia (4/46, 8.7%) and speech disturbances (2/46, 4.3%). 17/46 patients (37.0%) presented with highly symptomatic disease.

### Ki67 proliferation index in pilocytic astrocytoma patients

The Ki67 proliferation index could be evaluated in 35/46 (76.1%) patients, with a median of 2.8% (range: 0.5–13.4%). No correlation between age at diagnosis and Ki67 proliferation index was observed (Spearman’s r = − 0.041, p = 0.814).

### Tumor location and resectability in adult pilocytic astrocytoma patients

**Fig. 1:**
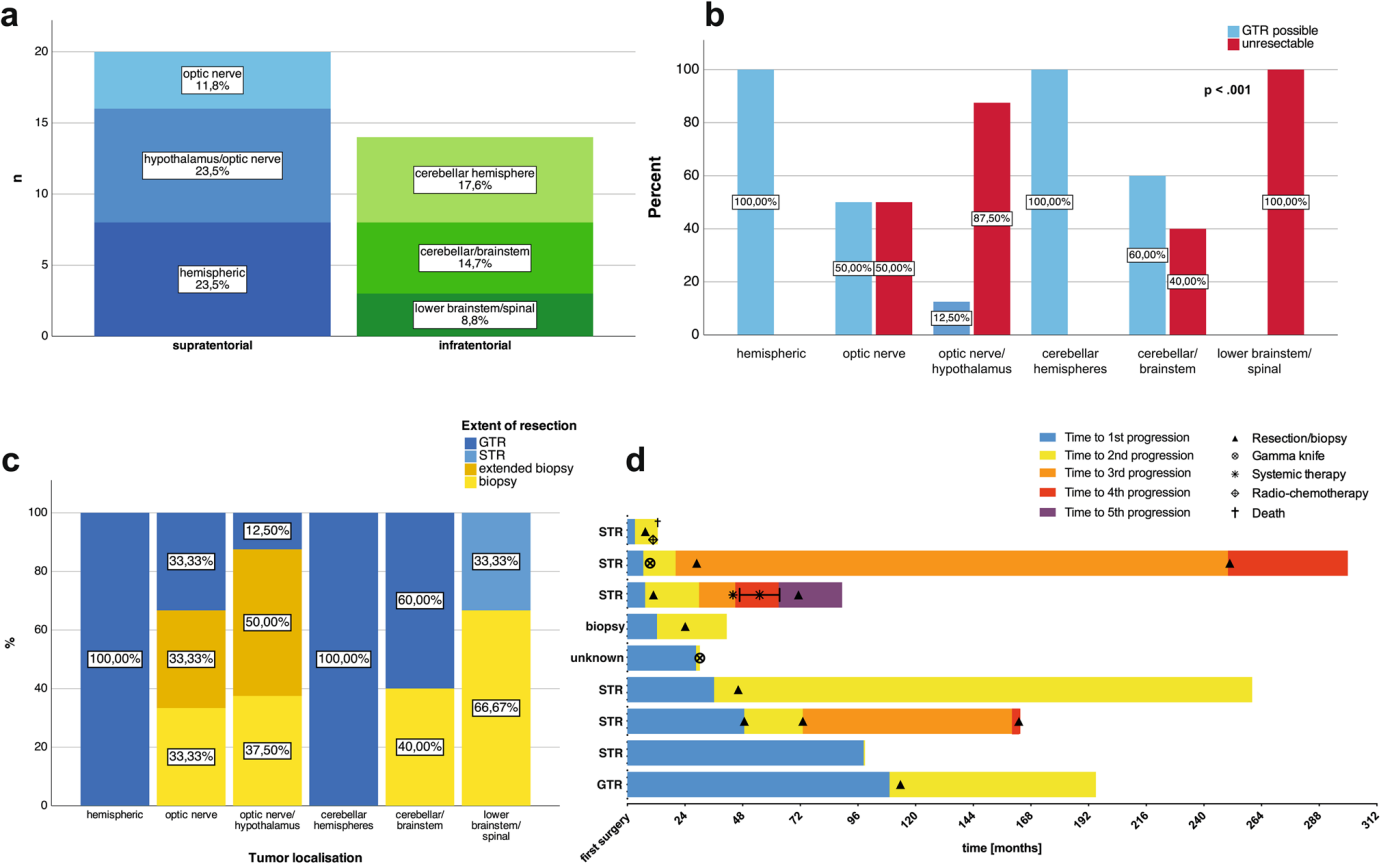
Clinical characteristics of the described cohort. **a** Tumor localization. Percentages are based on the cohort with available preoperative imaging (n = 34). **b** Resectability according to different tumor localisations (p < 0.001, Fisher’s exact test). **c** Extents of resection according to different tumor localisations. **d** Clinical course in patients with tumor progression after first resection. Extents of resection are given as estimated by the resecting neurosurgeon. Timelines start at time of first surgery and end at last follow-up or death, unless specified otherwise

Preoperative imaging was available for further analysis in 34/46 (73.9%) patients, as in the other patients preoperative imaging was performed not in the center and therefore not available for the analysis. 20/34 (58.8%) patients presented with supratentorial location, while infratentorial lesions were observed in 14/34 (41.2%) patients. Among the supratentorial located lesions, 8/34 (17.4%) tumors were located in the optic nerve with hypothalamic involvement, 4/34 (17.4%) in the optic nerve without hypothalamic involvement and 8/34 (17.4%) in the cerebral hemispheres. Most infratentorial lesions were located in the cerebellar hemispheres (6/34, 17.6%), followed by cerebellar lesions with involvement of the brainstem (5/34, 14.7%) and lower brainstem/spinal lesions (3/34, 8.8%, Fig. 1a). Location did not correlate with symptomatic burden (p = 0.937, Fisher’s exact test).

Based on pre-operative imaging, resectability could be assessed in 34/46 (73.9%) patients. Gross total resection (GTR) was judged possible in 20/34 (58.8%) patients, while 14/34 (41.2%) lesions were already pre-surgically classified as unresectable. All tumors in the cerebral and cerebellar hemispheres, 3/5 (40.0%) tumors located in the cerebellar/brainstem, 2/4 (50.0%) tumors in the optic nerve and 1/8 (12.5%) in the optic nerve with hypothalamic involvement were classified as resectable. All tumors in the lower brain stem/spinal region were assessed as unresectable. In consequence, tumor localization correlated with resectability (p < 0.001, Fisher’s exact test, Fig. 1b).

### Clinical course of pilocytic astrocytoma patients

All patients underwent histological confirmation of PA either by primary resection or biopsy. Postoperative imaging for the evaluation of the extent of resection was available in 32/46 (69.6%) patients. GTR was achieved in 18/32 (56.3%) patients. In 2 patients, GTR was possible based on pre-operative imaging but no postoperative imaging to judge the extent of resection was available. STR was achieved in 1/32 (3.1%) patients. An extended biopsy was performed in 5/32 (15.6%), while stereotactic biopsies were obtained in 8/32 (25.0%) patients. Extents of resection according to tumor location are given in Fig. 1c. GTR could be achieved in 18/18 cases where the lesion had been classified as resectable based on preoperative imaging. In none of the patients undergoing resection, invasion of tumor tissue into neighboring structures was documented in surgery reports. None of the included patients received adjuvant radiotherapy or chemotherapy after surgery.

Local recurrence of totally resected PA (as estimated by the resecting neurosurgeon) was seen in 1/25 (4%) patients where GTR was performed, while progression of incompletely resected or biopsied PA (as documented in surgery reports) was observed in 7/18 (38.9%) patients. 3 patients experienced 2 or more tumor recurrences/progressions. One patient was lost to follow-up immediately after the diagnosis of tumor recurrence. The clinical course of patients with documented tumor progression is illustrated in Fig. 1d.

Treatment was initiated in all patients with PA recurrence/progression. 6/8 (75.0%) patients with recurrence/progression were re-resected and STR was achieved in 5/8 (62.5%) patients, whereas one patient was biopsied only. 2/8 (25.0%) patients were treated with gamma knife. Systemic therapy was applied in 2/9 (22.2%) patients as a salvage treatment after PA recurrence/progression. The used agents were vinblastine and bevacizumab in one patient and combined, temozolomide-based radio-chemotherapy in the patient diagnosed with glioblastoma in the recurrence setting.

In 2/9 (22.2%) patients with PA recurrence/progression the histological characteristics of the recurrent/progressive specimen differed from the initial diagnosis of PA. Recurrent/progressive tumor tissues were histologically classified as glioma (not otherwise specified) and glioblastoma in one patient each. Based on DNA methylation, histological classification was revised in both patients. Although initial diagnosis of PA was confirmed by the methylation classifier, the recurrent sample was classified as of glioma, not otherwise specified (NOS). In the second patient with changes in the histological characteristics, methylation classification yielded the diagnosis of anaplastic astrocytoma with piloid features.

### Outcomes and survival analysis

**Fig. 2:**
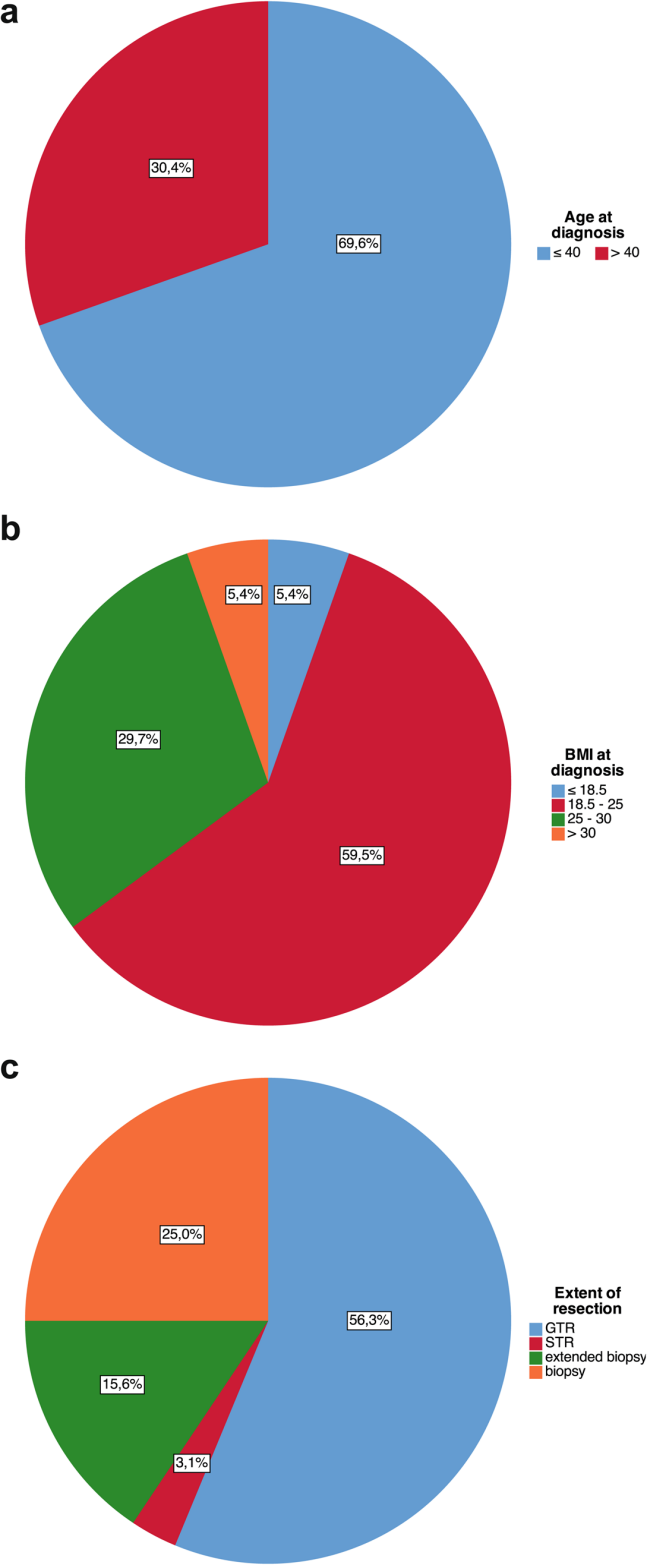
Distribution of risk factors associated with prognosis in our cohort. **a** Age ≤ and > 40 years at diagnosis in the entire cohort (n = 46). **b** BMI (where available, n = 38). **c** Extents of resection as determined by postoperative imaging (n = 32, postoperative imaging not available in 14/46 patients)

**Fig. 3:**
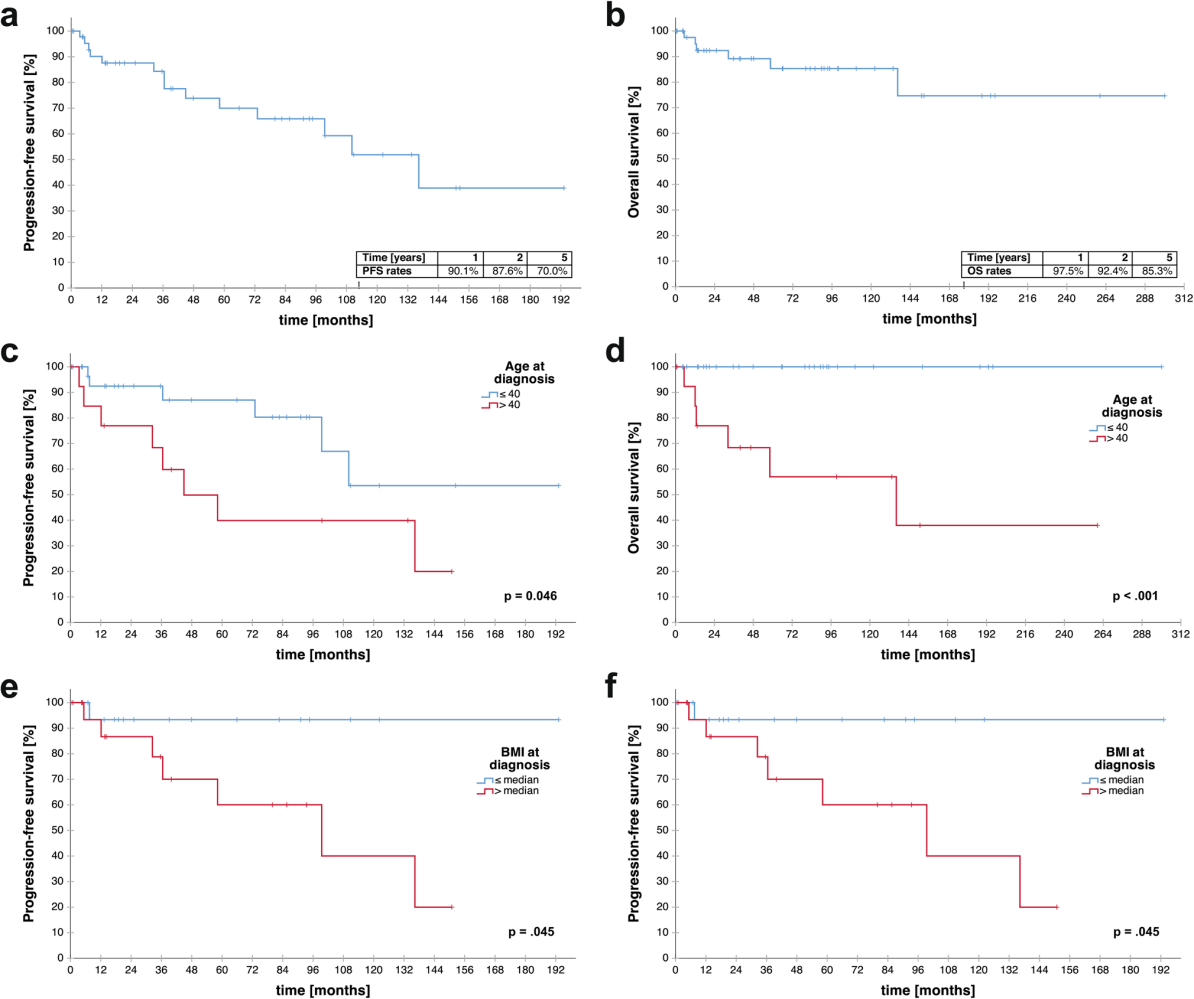

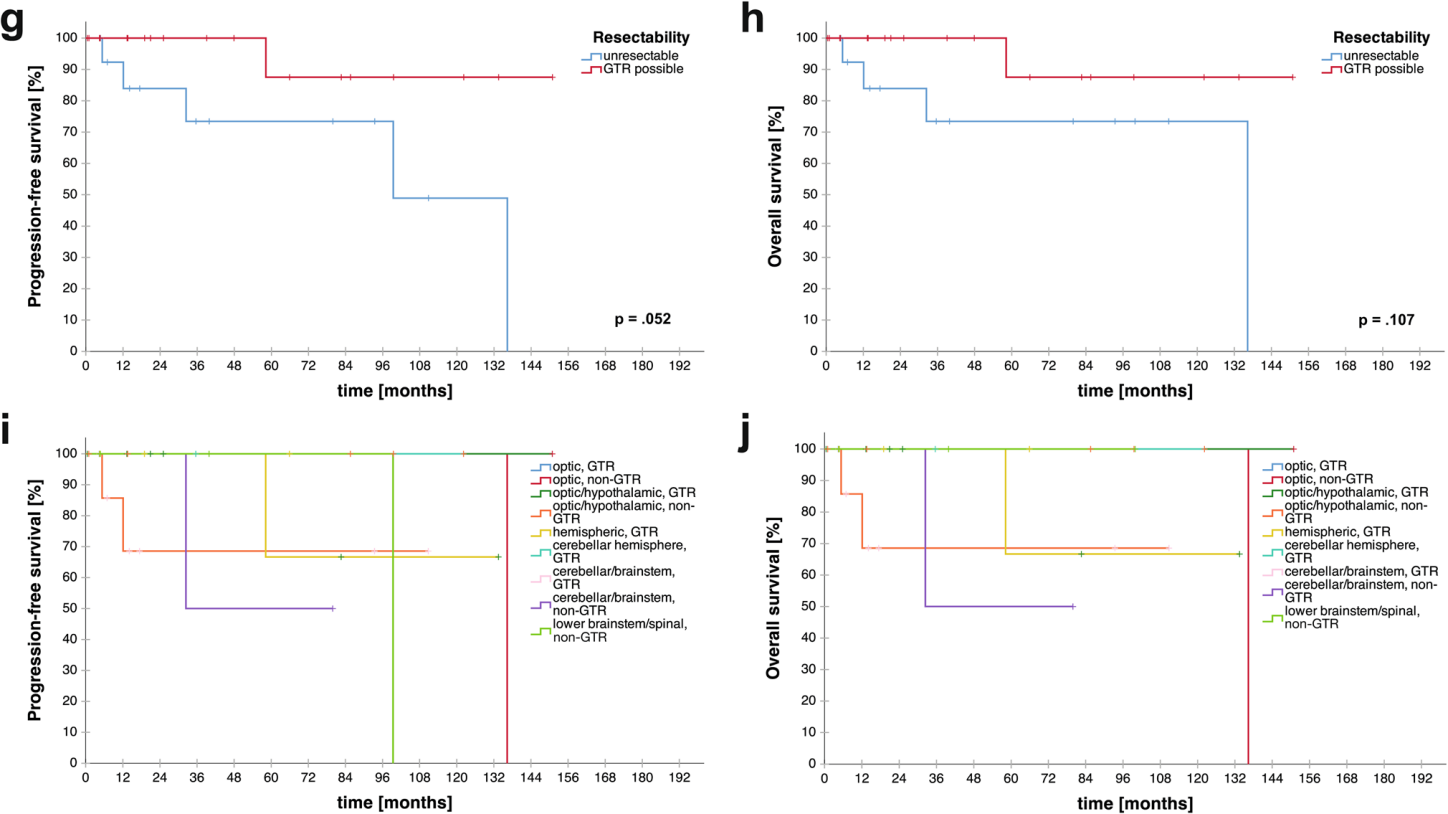
Overall and progression-free survival analysis. **a** PFS and **b** OS of the entire cohort. **c**, **d** PFS/OS according to age at first diagnosis (≤ 40 versus > 40 years). **e**, **f** PFS/OS according to BMI at first diagnosis (≤ median versus > median in the cohort). **g**, **h** PFS/OS according to resectability (GTR achievable vs. unresectable). **i**, **j** PFS/OS according to tumor site and extent of resection. p-values are given as determined by the log-rank test

The distribution of risk factors associated with prognosis in our cohort is illustrated in Fig. 2. Median PFS was 136.3 months and median OS was not reached after a median follow up of 53.0 months (Fig. 3a, b). Survival rates at 5 years were 85.3% and 70.0% for OS and PFS, respectively.

Univariate survival analyses are given in Table 2. Patients with an age over 40 had significantly shorter PFS (median PFS: 44.9 vs. n.r., p = 0.046, log-rank test; Fig. 3c) and OS (136.3 vs. not reached; p < 0.001; log-rank test; Fig. 3d) than younger individuals. Further, patients with a BMI higher than the median (> 22.84 kg/m^2^) had shorter PFS (PFS 99.4 months vs. n.r.; p = 0.045, log-rank test, Fig. 3e) and OS (OS 136.3 months vs n.r.; p = 0.038; log-rank test; Fig. 3f).

**Table 2:** Univariate survival analysis

Survival analysis (univariate)		Median PFS [months]	p value (log rank test)	Median OS [months]	p value (log rank test)
Gender	Male	73.0	0.060	n.r	0.240
	Female	136.3		136.3	
Age	≤ 40	n.r	**0.046**	n.r	** < 0.001**
	> 40	44.9		136.3	
BMI	≤ median	n.r	**0.045**	n.r	**0.038**
	> median	99.4		136.3	
KPS	> 80	n.r	0.804	n.r	0.435
	≤ 80	136.3		n.r	
Symptoms	≤ 2	n.r	0.729	n.r	0.736
	> 2	136.3		n.r	
Ki-67 proliferation index	≤ 2.6%	n.r	0.400	n.r	0.600
	> 2.6%	n.r		n.r	
Dexamethasone use at first diagnosis/resection	Yes	99.4	0.134	n.r	0.448
	No	n.r		n.r	
Tumor location	Supratentorial	136.3	0.945	136.3	0.509
	Infratentorial	n.r		n.r	
Resectability	GTR achievable	n.r	0.052	n.r	0.107
	Unresectable	99.4		136.3	

p values in bold indicate numbers below the level of significance (p < 0.05)

*PFS* progression-free survival, *OS* overall survival, *n.r.* not reached, *BMI* body mass index, *KPS* Karnofsky Performance Scale, *GTR* gross total resection, *STR* subtotal resection

Of note, Ki67 proliferation index did not correlate with PFS (p = 0.400) and OS (p = 0.600).

Although not statistically significant, resectability was tendentially associated with higher PFS (n.r. vs. 99.4 months, p = 0.052, Fig. 3g) and OS (n.r. vs. 136.3 months, p = 0.107, Fig. 3h) as patients with unresectable lesions presented with a numerically shorter PFS and OS. Survival plots with respect to extent of resection and tumor location are given in Fig. 3i, j for PFS and OS, respectively. Median PFS was only reached in cerebellar/brain stem, non-GTR (79.9 months), lower brainstem/spinal, non-GTR (99.4 months) and optic, non-GTR (136.3 months) patients. Median OS was only reached in cerebellar/brain stem, non-GTR (79.9 months) and optic, non-GTR (136.3 months) patients. Statistical analysis was omitted due to small sample sizes.

## Discussion

PA is a rare disease in adults, resulting in only limited knowledge on the clinical course and risk factors for tumor recurrence or progression. Here, we investigated the clinical characteristics in a relatively large cohort of adult PA patients and observed higher recurrence/progression rates in the adult population as compared to the previously reported ones in pediatric patients. Clinical factors such as higher age and higher BMI were associated with impaired prognosis in adult PA patients and may be useful in the prognostic assessment of adult PA patients.

The recurrence/progression rate in the present adult PA cohort was 19.6% and thereby notably higher than the average recurrence rate of 10% reported in pediatric PA patients [12]. Indeed, also the median PFS time of 136.3 months was shorter than the one reported in children with PA [13]. Previous series including 10 to 127 adult PA patients reported median PFS times ranging from 16.5 months to > 178.8 months [4, 6, 14–18]. In the present cohort, age < 40 years was associated with improved PFS and OS. Clinical aggressive courses including leptomeningeal dissemination [19, 20], drop metastases [21] and even skeletal metastases were reported more frequently in adult PA patients [22]. The differences in the clinical course of adult and pediatric PA patients might be caused by different biological backgrounds [23, 24]. PI3K/AKT pathway alterations were more frequently observed in a cohort containing also adult patients and were associated with signs of anaplasia as well as with a clinically more aggressive behavior [24]. In the Surveillance, Epidemiology and End Results (SEER) program study, adults performed significantly worse compared to pediatric patients, even if cancer-specific survival was chosen as endpoint instead of all-cause mortality [7]. Despite the knowledge of increased recurrence risk in adult patients, the therapeutic concept of pediatric and adult patients does currently not differ. As in other glioma subtypes, gross-total resection was tendentially associated with improved prognosis, supporting that maximum safe neurosurgical resection should be performed in every patient with PA whenever possible. Of note, neurosurgical resectability and extent of resection depends on tumor site and invasion of the tumor into surrounding structures. In our cohort, all tumors located in the cerebral and cerebellar hemispheres were classified as resectable based on preoperative imaging, while all tumors in the lower brain stem were unresectable due to complex tumor location.

Adjuvant radiation after subtotal resection remains controversial, as the only so far conducted clinical trial did not include patients over 50 years and concentrated on patients with very favorable prognostic factors [25]. Other retrospective series indicate an added clinical benefit of radiation in case of unfavorable prognosis, however based on the limited available data, treatment decisions have to be made on an individual basis and should include tumor- and patient-specific parameters for prognostic assessment [17].

Histological transformation to diffuse lower-grade glioma or glioblastoma was observed in our adult PA cohort. Similar cases of histological transformation were reported in both children and adults [26–30]. As the molecular drivers of transformation remain unknown, actual misdiagnosis due to impaired diagnostic possibilities and close histological resemblance of CNS tumors has to be considered [7]. The morphology-concentrated diagnostic approach in neuropathology is challenged by similar features in small tumor samples. Inclusion of objective molecular analysis is increasingly applied to ensure precise diagnosis [2, 10]. Here, methylation profiles were used to address these limitations of morphology-based diagnosis in order to provide a more accurate, objective diagnostic tool, allowing diagnosis also in smaller samples or in samples with unspecific histologic features [10]. Methylation profiles were shown to identify distinct CNS tumor types, correlate with survival prognosis and even identified new tumor subtypes based on particular methylation profiles [9, 31]. Indeed, in the present cohort re-classification using the methylation pattern supported the initial PA diagnosis in the patient with histological change to glioma, not otherwise specified (NOS). In another patient, the diagnosis was reclassified to anaplastic astrocytoma with piloid features, as it was also observed previously where histological diagnoses of PA were reclassified to anaplastic PA, ependymoma, rosette-forming glioneuronal tumor or even diffuse midline glioma (H3K27M-mutant) or IDH-wildtype glioblastoma [10], underscoring the additional diagnostic potential of methylation classification in selected cases.

Higher BMI at diagnosis was statistically associated with impaired PFS and OS in the present PA cohort. Higher BMI was previously reported as an independent prognostic factor in various extracranial cancer entities [32, 33] and in the context of brain tumors, as higher pre-diagnostic BMI was associated with impaired survival prognosis in patients suffering from high-grade glioma [34, 35]. The complex metabolic condition of obesity associated with hyperglycemia, insulin resistance and elevated release of fatty acids might promote growth signals resulting in enhanced tumor growth [36]. Further, adipose tissue-derived hormones (adipokines) might impact the immune system and thereby enhance tumor growth, further adding to the multiple interactions of obesity and tumor growth [37]. With regard to metabolic comorbidities, 3 patients in our cohort had a history of type 2 diabetes mellitus and 1 of them was diagnosed with hyperlipidemia, while no other metabolic conditions were reported in our cohort. As a proof of concept, calorie-restricted ketogenic diet was associated with tumor control in a pre-clinical glioblastoma model, supporting that metabolic conditions can impact CNS tumor cell growth [38]. However, given the potentially harmful consequences of diets during a cancer treatment, controlled exercise rather than dietary restrictions should be considered as an addition to the overall treatment plan [39, 40].

Certainly, the limitations of a retrospective single-center analysis have to be considered in the interpretation of the present data. Furthermore, due to the low patient number, multivariate survival analyses could not be performed. In addition, MRI data at diagnosis was not available for the whole cohort. Methylation analysis of cases with a change of histology at recurrence was not possible at initial samples due to low tumor cell content. Further in-depth investigation of driver mutations including the BRAFV600E status would be of interest, however we concentrated on clinical parameters as routine testing of BRAFV600E mutation was not available in out cohort. However, due to the rare occurrence of PA in adults, there are only limited reports describing the clinical course of PA in a non-pediatric cohort. Indeed, with 46 patients available for analysis, the present cohort is among the largest published so far. In contrast to previous studies, also patients > 50 years could be included in the analysis providing an important, clinically well characterized cohort to study prognostic parameters in adult PA patients. The population-based Surveillance, Epidemiology and End Results (SEER) program study could include as much as 865 patients, however the amount of clinical data is limited and the study therefore mainly concentrated on survival. Therefore, the observed correlations should be validated in an independent, ideally prospective multi-centric cohort.

## Conclusion

Our study provides further insights into the clinical course of PA in adults treated at a tertiary care center. PA in adults seem to be characterized by a more aggressive clinical course compared to PA in pediatric patients. Malignant transformation can be observed occasionally and might impact the impaired prognosis of adult PA patients. We further support the importance of maximal safe resection in PA patients as non-resectable cases as determined by tumor location presented with higher recurrence/progression rates. BMI and age were associated with survival prognosis and might be considered for prognostic assessment of adult PA patients.
